# Mesotheliomata in Rats after Inoculation with Asbestos and Other Materials

**DOI:** 10.1038/bjc.1973.134

**Published:** 1973-08

**Authors:** J. C. Wagner, G. Berry, V. Timbrell

## Abstract

**Images:**


Br. J. Cancer (1973) 28, 173

### MESOTHELIOMATA IN RATS AFTER INOCULATION WITH ASBESTOS AND OTHER MATERIALS

J. C. WAGNER, G. BERRY AND V. TIMBRELL

*From the Medical Research Council's Pneumoconiosis Unit, Llandough Hospital, Penarth, Glamorgan*

Received 21 March 1973. Accepted 10 April 1973

**Summary.—Four experiments in which SPF Wistar rats were inoculated intrapleurally with asbestos or other materials are described. Mesotheliomata were observed in a considerable proportion of animals with all the samples of asbestos used and with a sample of brucite. A few were produced with synthetic aluminium silicate fibres and single ones with barium sulphate, glass powder and aluminium oxide. The risk of developing a mesothelioma at a given time after injection was approximately proportional to the dose. Of the UICC standard reference samples, crocidolite was the most carcinogenic and removal of the oils by benzene extraction did not alter the carcinogenicity of these samples. Chemical properties also seem unlikely to be the main factor producing mesotheliomata but the results support the hypothesis that the finer fibres are the more carcinogenic, and this is additional to the known aerodynamic advantage which the finer fibres have in penetrating to the periphery of the lung.**

We report here the results of 4 experiments in which asbestos and other test materials were administered to rats by intrapleural inoculation. These experiments were planned to obtain more information on the carcinogenic effect of asbestos and other materials than could be obtained from our original 2 experiments (Wagner and Berry, 1969). Preliminary results of some of the present experiments were given by Wagner, Berry and Timbrell (1970), Wagner (1970, 1972). In this paper the complete results are given, with emphasis on the light they throw on the aetiology of mesotheliomata, taking into account the oils and waxes present in asbestos, other chemical characteristics and the physical characteristics.

### MATERIALS AND METHODS

In all 4 experiments specific pathogen-free (SPF) rats, of the Wistar strain were used. These rats had been bred at the Unit from stocks given to us by Imperial Chemical Industries, Pharmaceutical Division at Alderley Edge, Cheshire in 1964 and 1968.

The following materials were used:

1. *SFA chrysotile.*—A super fine sample obtained from a Canadian mine, and produced by water sedimentation separation from grade 7, the most fully milled commercial product.
2. *Crocidolite.*—Prepared from virgin fibre from a mine in the North West Cape. Both (1) and (2) were from the same samples as used in the earlier experiments (Wagner and Berry, 1969).
3. *UICC Standard reference samples.*—Samples of amosite, anthophyllite, Canadian chrysotile, Rhodesian chrysotile and crocidolite (Timbrell, Gilson and Webster, 1968) prepared following recommendations of *l'Union Internationale Contre le Cancer* (UICC).
4. *Benzene-extracted UICC Standard reference samples.*—Samples of (3) which had been repeatedly extracted for 64 hours by hot benzene using a Soxhlet apparatus to remove oils and other benzene-soluble substances. After extraction the benzene was first allowed to evaporate naturally and finally the samples were warmed to 80°C for 24 hours to remove any remaining benzene. After this treatment the samples were tested for the presence of any residual benzene by extracting test portions with cyclohexane and examining the solutions by means of ultraviolet spectrophotometry; no benzene was detected in these solutions.

5. *Canadian chrysotiles*.—Samples from 8 mines (A, B, . . . H) in Canada. These were the same samples used to prepare the UICC standard reference sample of Canadian chrysotile (Timbrell and Rendall, 1971) but were milled for our purpose more finely than the reference sample.

6. *Brucite*.—A specimen of brucite, which however also contained chrysotile. This specimen was from Canadian mine H and consisted of long coarse brownish fibres above 50 cm in length. The sample was milled to respirable particle size.

7. *Barium sulphate*.—Used as a control. This was prepared in the laboratory by the addition of sulphuric acid to barium chloride solution.

8. *Saline*.—Sterile physiological saline was also used as a control.

9. *Ceramic fibre*.—A synthetic aluminium silicate fibre. This fibre was prepared for experimental use by grinding in a ceramic ball mill and extracting the respirable fraction by settlement in air. The fibre diameters were between 0·5 and 1 $\mu$m.

10. *Fibreglass*.—A borosilicate. The nominal diameters of the fibres were between 1·5 and 2·5 $\mu$m but in fact only 30% were within this range, the range extending to 7 $\mu$m. The sample was prepared by embedding the fibres in water soluble wax, chopping in a microtome and washing away the wax. Over 60% of the fibres were longer than 20 $\mu$m.

11. *Glass powder*.—A borosilicate all in the respirable range (less than 8 $\mu$m projected area diameter).

12. *Aluminium oxide*.—A non-fibrous material all in the respirable range (less than 10 $\mu$m projected area diameter).

13. *SFA chrysotile* (Second sample).—A sample from the same mine and prepared similarly to (1), but taken several years later.

*Experiment* 1—*Varying dose*

There were 5 doses, 0·5, 1, 2, 4 and 8 mg per rat, of SFA chrysotile and crocidolite. There were about 12 rats per dose per dust and inoculation was during March 1965.

*Experiment* 2.—*Canadian chrysotiles*

The experimental materials were 7 of the 8 Canadian chrysotile samples, SFA chrysotile and saline control. The dose was 20 mg per rat. There were 16 rats for each Canadian sample, 32 for SFA chrysotile and 48 controls and inoculation was during December 1966.

*Experiment* 3—*UICC samples and Canadian chrysotiles*

The materials used were the 5 UICC reference samples both in the normal and oil-free forms, the 8 Canadian chrysotile samples, brucite and barium sulphate and saline controls. The dose was 20 mg per rat. There were 24 rats for each of the Canadian samples and 32 for each of the other treatments. Inoculation took place between November 1967 and February 1968.

*Experiment* 4.—*Various dusts*

The materials injected were ceramic fibre, fibreglass, glass powder, aluminium oxide, SFA chrysotile and also the second sample of SFA chrysotile. The dose was 20 mg per rat and there were up to 36 rats per treatment (because of a shortage of animals it was not possible to allocate 36 to all treatments and in addition inoculation fatalities could not be replaced). Inoculation took place in June and July 1969.

For each experiment animals were allocated at random to treatments. The age of the rats at inoculation was about 6 weeks for Experiments 1, and 3 and 13 weeks for Experiments 2 and 4. In Experiments 1 and 3 there were equal numbers of male and female rats, whilst in Experiment 2 there were 3 times as many females as males, and in Experiment 4 there were twice as many males as females.

*Methods*

The experimental materials were made up in a suspension of physiological saline with a concentration of 50 mg/ml for Experiments 2, 3 and 4 and for Experiment 1 the concentration was such that the required dose would be present in 0·4 ml of suspension. The rats were anaesthetized with ether and a needle attached to a two-way tap was then introduced into the right axilla at the level of the second nipple. One arm of the two-way tap was attached to a capillary manometer, which gave a negative reading when the needle reached the pleural cavity. Details of the method of inoculation were given by Wagner and Berry (1969). Following injection the rats were caged in fours isolated in a special unit. They were fed on a proprietary brand of autoclaved cubes, and water *ad libitum*. Each rat was allowed to live until it died or appeared to be distressed and a full necropsy examination was carried out, except for a few which had been cannibalized.

The results have been analysed using the model given by Pike (1966) and shown to be valid for experiments of this type (Berry and Wagner, 1969). Fuller details are given in in the appendix, and it need only be noted that this method of analysis allows a constant $c$ to be estimated for each of the treatment groups and that this constant, which we will refer to as the " carcinogenicity factor ", serves as a single index summarizing the mesothelioma experience of each group. It combines the information on the proportion of animals which developed a mesothelioma with the times after inoculation at which the mesotheliomata occurred. Also, since the method of estimation eliminates mortality due to other causes, chance variations in natural mortality between different treatment groups do not affect the treatment comparisons, nor do systematic differences in natural mortality between different experiments, such as that resulting from the animals in Experiments 2 and 4 being older than those in Experiments 1 and 3, affect comparisons between experiments.

Where significance levels are quoted they are usually based on the *chi*-squared approximation to a likelihood-ratio test.

### RESULTS

There were 13 rats for which histological examination was not possible, leaving 1112 rats included in the results.

The predominant finding was that a high proportion of most asbestos treated groups developed mesotheliomata and the results will be given mainly in terms of the number of mesotheliomata and the time when they occurred. Some details of the results are given in Tables I–IV. A total of 386 mesotheliomata occurred but the histological features of these tumours will not be described here as there is nothing to add to the features described for the original experiments (Wagner and Berry, 1969). Also, in the presentation and analysis of the results, no account has been taken of the sex of the rats. The original experiments show males and females equally likely to develop a mesothelioma, and the present experiments confirm this.

### *Experiment* 1

There is a relationship between the number of mesotheliomata and the dose for both SFA chrysotile and crocidolite. This implies that the carcinogenicity is related to dose ($d$) and we considered this relationship in the form of the carcinogenicity factor being proportional to a power of dose, *i.e.* $c = bd^p$ where $b$ and $p$ are constants. The power $p$ was estimated separately for each dust, giving 0·73 for chrysotile and 0·96 for crocidolite. Because of the small number of mesotheliomata, however, these estimates are not very precise and the approximate 95% limits are 0·3–1·3 and 0·2–1·9 for chrysotile and crocidolite respectively. There

TABLE I.—*Experiment* 1 *Results*

| | | Number of rats with histology | Number with a mesothelioma | Survival time (days) of first mesothelioma | Mean survival (days) |
|---|---|---|---|---|---|
| SFA chrysotile | 0·5 mg | 12 | 1 | 512 | 784 |
| SFA chrysotile | 1 mg | 11 | 3 | 615 | 729 |
| SFA chrysotile | 2 mg | 12 | 5 | 425 | 664 |
| SFA chrysotile | 4 mg | 12 | 4 | 470 | 762 |
| SFA chrysotile | 8 mg | 12 | 8 | 496 | 692 |
| Crocidolite | 0·5 mg | 11 | 1 | 992 | 809 |
| Crocidolite | 1 mg | 12 | 0 | — | 760 |
| Crocidolite | 2 mg | 12 | 3 | 562 | 777 |
| Crocidolite | 4 mg | 13 | 2 | 917 | 819 |
| Crocidolite | 8 mg | 11 | 5 | 799 | 689 |

TABLE II.—*Experiment* 2 *Results*

| | Number of rats with histology | Number with a mesothelioma | Survival time (days) of first mesothelioma | Mean survival (days) |
|---|---|---|---|---|
| Canadian chrysotile A | 16 | 8 | 416 | 642 |
| Canadian chrysotile B | 16 | 10 | 416 | 594 |
| Canadian chrysotile C | 16 | 5 | 488 | 702 |
| Canadian chrysotile D | 16 | 10 | 461 | 624 |
| Canadian chrysotile E | 16 | 7 | 405 | 573 |
| Canadian chrysotile F | 16 | 10 | 421 | 619 |
| Canadian chrysotile H | 16 | 4 | 384 | 602 |
| SFA chrysotile | 32 | 22 | 376 | 553 |
| Control | 48 | 0 | — | 728 |

TABLE III.—*Experiment* 3 *Results*

| Material | Number of rats with histology | Number with a mesothelioma | Survival time (days) of first mesothelioma | Mean survival (days) |
|---|---|---|---|---|
| *UICC samples* | | | | |
| Amosite | 32 | 12 | 377 | 716 |
| Amosite benzene-extracted | 32 | 11 | 590 | 718 |
| Anthophyllite | 32 | 8 | 498 | 761 |
| Anthophyllite benzene-extracted | 32 | 14 | 533 | 728 |
| Chrysotile (Canadian) | 32 | 10 | 541 | 747 |
| Chrysotile (Canadian) benzene-extracted | 32 | 9 | 632 | 753 |
| Chrysotile (Rhodesian) | 31 | 7 | 502 | 693 |
| Chrysotile (Rhodesian) benzene-extracted | 32 | 5 | 659 | 686 |
| Crocidolite | 32 | 19 | 586 | 682 |
| Crocidolite benzene-extracted | 30 | 19 | 468 | 657 |
| Canadian chrysotile A | 24 | 14 | 488 | 712 |
| Canadian chrysotile B | 22 | 9 | 437 | 636 |
| Canadian chrysotile C | 24 | 9 | 460 | 717 |
| Canadian chrysotile D | 23 | 12 | 534 | 669 |
| Canadian chrysotile E | 24 | 9 | 489 | 660 |
| Canadian chrysotile F | 24 | 13 | 484 | 675 |
| Canadian chrysotile G | 23 | 16 | 576 | 659 |
| Canadian chrysotile H | 23 | 14 | 429 | 663 |
| Brucite | 32 | 18 | 502 | 680 |
| Barium sulphate | 30 | 1 | 436 | 783 |
| Saline control | 32 | 0 | — | 818 |

TABLE IV.—*Experiment* 4 *Results*

| | Number of rats with histology | Number with a mesothelioma | Survival time (days) of first mesothelioma | Mean survival (days) |
|---|---|---|---|---|
| Ceramic fibre | 31 | 3 | 743 | 736 |
| Fibreglass | 35 | 0 | — | 774 |
| Glass powder | 35 | 1 | 516 | 751 |
| Aluminium oxide | 35 | 1 | 646 | 710 |
| SFA chrysotile | 36 | 23 | 325 | 568 |
| SFA chrysotile (2nd sample) | 32 | 21 | 382 | 639 |

are some theoretical grounds for choosing $p$ to be an integer and therefore $p$ was taken as unity. The values of the carcinogenicity factor adjusted to a dose of 20 mg were then $4{\cdot}10 \times 10^{-9}$ for chrysotile and $1{\cdot}70 \times 10^{-9}$ for crocidolite.

*Experiments* 2, 3 *and* 4

Comparing first the effects of the separate Canadian samples (Table V), there is considerable variation between Experiments 2 and 3 and this is mainly because of the small number of animals in

TABLE V.—*Estimates of Carcinogenicity Factor* ($\times 10^9$) *for Experiments* 2, 3 *and* 4

| | Experiment | | |
|---|---|---|---|
| | 2 | 3 | 4 |
| Canadian chrysotile A | 1·25 | 1·20 | — |
| Canadian chrysotile B | 2·23 | 1·16 | — |
| Canadian chrysotile C | 0·68 | 0·67 | — |
| Canadian chrysotile D | 2·39 | 1·23 | — |
| Canadian chrysotile E | 1·89 | 0·97 | — |
| Canadian chrysotile F | 1·90 | 1·40 | — |
| Canadian chrysotile G | — | 2·09 | — |
| Canadian chrysotile H | 1·10 | 1·84 | — |
| SFA chrysotile | 4·72 | — | 2·85 |
| SFA chrysotile (2nd sample) | — | — | 2·28 |
| Brucite | — | 1·21 | — |
| Barium sulphate | — | 0·04 | — |
| Ceramic fibre | — | — | 0·16 |
| Glass powder | — | — | 0·04 |
| Aluminium oxide | — | — | 0·05 |

each group. There was overall about 30% more carcinogenicity in Experiment 2 than in Experiment 3 but the difference is not significant ($P > 0{\cdot}1$). Sample C has the lowest carcinogenicity in each experiment and overall is significantly the least carcinogenic ($P < 0{\cdot}05$) but apart from this no differences between the samples were detected. These samples have been analysed for certain metals (Holmes, Morgan and Sandalls, 1971; Morgan and Cralley, 1973) and in Table VI the results of these analyses are shown, together with the carcinogenicity factor obtained by combining the 2 experiments. The correlation coefficients between the carcinogenicity factor and the different metals are −0·13 for iron, −0·58 for chromium, 0·04 for cobalt, −0·02 for nickel, −0·39 for scandium and −0·04 for manganese. None of these is significant and it is reasonably clear from examination of the chemical properties of sample C that the low carcinogenicity of this sample is not because of a low content of any of these metals.

Turning now to the UICC reference samples (Table VII), there are no significant differences between the normal and benzene-extracted samples, and overall 58 mesotheliomata occurred with the benzene-extracted samples and 56 with the untreated samples. Crocidolite was the most carcinogenic sample with the others in order amosite, anthophyllite, Canadian chrysotile and Rhodesian chrysotile. The difference between the two samples of chrysotile is not significant. Holmes *et al.* (1971) also carried out chemical analyses on the UICC reference samples. There were very large differences between the samples in the amount of the different metals present and it is clear that these bear no relation to the carcinogenicity.

The sample of brucite proved as carcinogenic as the Canadian samples of chrysotile (Table V). Non-asbestos materials which produced the occasional mesothelioma were ceramic fibre, barium sulphate, glass powder and aluminium oxide.

The second sample of SFA chrysotile proved similar in carcinogenic effect to the original sample.

### DISCUSSION

The application of the test materials by intrapleural inoculation may be criticized as unrealistic in comparison with human exposure, about which the animal experiments are intended to provide relevant information. Nevertheless, this type of experiment has an important part to play. With an inhalation experiment, which provides a realistic route of entry of the test material, there are 2 factors involved. First, the penetration of dust through the airways and alveoli will differ with different samples of dust (Timbrell, 1965). The second factor is the effect of the dust, given that it has reached the pleura. In inoculation experiments only the second factor is relevant and hence these experiments are simpler to interpret. This makes intrapleural inoculation a more suitable method for the investigation of questions such as whether extraction of the oils alters the carcinogenicity of an asbestos sample. The two types of experiment supplement one another and we will be reporting separately on 2 experiments in which rats were exposed to dust clouds of the UICC reference samples.

The varying dose experiment gave results which indicated that the risk of developing a mesothelioma at a given time after injection was proportional to the dose. This form of dose relationship was also found by Pike and Doll (1965) for lung cancer and smoking in man whereas Lee and O'Neill (1971) showed that after repeated applications of benzopyrene to the backs of mice the incidence rate of tumours was proportional to the square of the dose.

The carcinogenicity of the SFA chrysotile sample was similar in Experiments 1 and 2, after adjusting the former to a dose of 20 mg, and in Experiment 4 was lower but not significantly so. In all these 3 experiments the carcinogenicity of the SFA chrysotile was significantly greater than in the earlier experiment (Wagner and Berry, 1969) when the estimate of the carcinogenicity factor was $1{\cdot}68 \times 10^{-9}$. The crocidolite was also more carcinogenic in Experiment 1 than in the earlier experiment ($c = 1{\cdot}16 \times 10^{-9}$) but not significantly so. These differences could be the result of a change in susceptibility of the rats or of a change in the dust during storage.

The suggestion that natural oils and waxes (Harington, 1962), or contaminating oils from the preparation of the fibre (Harington and Roe, 1965; Roe, Walters and Harington, 1966) or from plastic storage bags (Commins and Gibbs, 1969) might contribute to the carcinogenicity of asbestos receives no support from our present experiments, which is in agreement with our original experiments (Wagner and Berry, 1969) when removal of the oils from the crocidolite sample resulted in no detectable change in carcinogenicity.

Harington and Roe (1965) also advanced the possibility that the presence of trace metals might be relevant to the carcinogenicity of asbestos. In our experiments with the Canadian samples the carcinogenicity was not related to the content of iron, chromium, cobalt, nickel, scandium or manganese. Also, the fact that all the types of asbestos, having very different chemical compositions, produce mesotheliomata makes it unlikely that the carcinogenicity of asbestos could be due to chemical properties.

Our experiments offer some evidence that the development of mesotheliomata is associated with the presence of fine fibrous material within the pleural cavity. First, UICC Canadian chrysotile is a mixture of batches of material from 8 Canadian mines, and the separate samples used were taken from the same batches (Timbrell and Rendall, 1971). The main difference in the subsequent preparation of the material was that the separate Canadian samples were ground more finely than the composite UICC sample. Comparing Tables V and VII, the carcinogenicities of all the separate Canadian samples were greater than that of the UICC Canadian chrysotile. Also, the samples of SFA chrysotile were from mine D. These were superfine samples and resulted in a very high carcinogenicity. It should be noted that of the Canadian samples the one with the lowest carcinogenicity (C) in both experiments was from a mine in British Columbia whilst the others were from 7 mines in the Quebec area. However, sample C could not be distinguished from the other samples by its size distribution.

A full quantitative analysis of our experimental results will only be possible when techniques are available for complete size characterization of the experimental materials, both before injection and present in the lungs at postmortem. Such techniques to determine the mass and the diameter and length distributions of the particles are being developed. However, even with the characterization methods at present available, a relationship emerges between the observed incidence of mesotheliomata and the physical factors.

A further factor that must be taken into account is the tendency of chrysotile fibres to fragment longitudinally into fine fibrils in lung fluids, the degree of frag-

TABLE VI.—*Carcinogenicity and Chemical Analysis of Canadian Samples—Experiments 2 and 3 Combined*

| Sample | Carcinogenicity factor (× 10⁹) | Iron (%) | Chromium parts/10⁶ | Cobalt parts/10⁶ | Nickel parts/10⁶ | Scandium parts/10⁶ | Manganese parts/10⁶ |
|---|---|---|---|---|---|---|---|
| G | 2·37 | 1·9 | 380 | 53 | 1400 | 7·5 | 420 |
| D | 1·70 | 4·0 | 930 | 78 | 1550 | 6·6 | 600 |
| F | 1·60 | 2·9 | 730 | 78 | 1900 | 7·7 | 450 |
| B | 1·59 | 3·4 | 780 | 110 | 2400 | 4·1 | 580 |
| H | 1·54 | 3·2 | 480 | 42 | 550 | 5·6 | 530 |
| E | 1·33 | 3·2 | 435 | 57 | 895 | 5·4 | 610 |
| A | 1·22 | 4·8 | 515 | 63 | 1150 | 5·0 | 540 |
| C | 0·67 | 2·0 | 1200 | 60 | 1800 | 12·0 | 420 |

TABLE VII.—*Estimates of Carcinogenicity Factor* ($\times 10^9$) *for UICC Reference Samples in Experiment* 3

| | Normal form | Benzene-extracted form |
|---|---|---|
| Amosite | 0·66 | 0·66 |
| Anthophyllite | 0·33 | 0·77 |
| Chrysotile (Canadian) | 0·50 | 0·42 |
| Chrysotile (Rhodesian) | 0·44 | 0·31 |
| Crocidolite | 1·45 | 1·87 |

mentation and hence the number of fibres and fibrils produced depending on the precise physical and physiological conditions. Amphibole types of asbestos, on the other hand, have characteristic fibre-diameter distributions which they appear to retain in lung tissue (Timbrell, Pooley and Wagner, 1970).

To illustrate this relationship, the electron micrographs of some of the materials are presented in decreasing order of their carcinogenicity (Fig. 1–8). For reasons given by Timbrell (1973), we shall consider as " significant " fibres those that are less than 0·5 $\mu$m in diameter and also greater than 10 $\mu$m in length. The non-chrysotile materials will be considered first. In the electron micrographs for UICC crocidolite (Fig. 2), UICC amosite (Fig. 4), UICC anthophyllite (Fig. 5), ceramic fibre (Fig. 7) and glass fibre (Fig. 8) it is evident that the number of " significant " fibres decreases with decreasing carcinogenicity of the materials. The glass fibre, for instance, contains long fibres but the majority of these are thicker than 0·5 $\mu$m. On the other hand, whereas a high proportion by weight of the brucite consists of large fibres, there are also present a number of very fine long fibrils. It is difficult to compare chrysotile samples (Fig. 1 and 6) with other types of material on this basis. But even so, the relative positions of the chrysotiles in the classification by carcinogenicity appears to correspond with the number of " significant " fibres present. For example, although the SFA chrysotile (Fig. 1) contains a high proportion by weight of non-fibrous particles, even before injection the fibres were in a highly dispersed state. The enormous number of fibres that complete fragmentation of chrysotile can produce will be clear from the illustration that a single fibre may fragment into 1000 fibrils. The fact that this SFA sample was the most carcinogenic of all the materials used corresponds to its highly dispersed state and its high content of " significant " fibres.

The above theory has been examined further using the results of Stanton and Wrench (1972). Their experiments were similar to ours and they used 17 samples, including several materials (UICC samples and glass fibres) after partial pulverization. They analysed their results by discounting submicroscopic fibrils and converting all longer fibres into microfibres of standard size (1·25 × 3·75 $\mu$m) on the assumption that fragmentation of both glass and asbestos occurred *in vivo*. The numbers of microfibres were then compared with the carcinogenicity of the materials. At first sight their results seem to conflict with our findings. But,

FIG. 1.—Electronmicrograph of SFA chrysotile (×1875).

FIG. 2.—Electronmicrograph of UICC crocidolite (×1875).

FIG. 4.—Electronmicrograph of UICC amosite (×1875).

FIG. 3.—Electronmicrograph of brucite (× 1875).

FIG. 5.—Electronmicrograph of UICC anthophyllite ($\times$ 1875).

FIG. 6.—Electronmicrograph of UICC Canadian chrysotile ($\times$ 1875).

FIG. 8.—Electronmicrograph of glass fibre (×1875).

FIG. 7.—Electronmicrograph of ceramic fibre (×1875).

when their materials were assessed in the manner used for our own samples, the order obtained by classification of the materials according to the number of "significant" fibres was in good agreement with the reported order of carcinogenicity. The smaller number of mesotheliomata observed from pulverized materials did not correlate with the estimated total number of particles that this pulverization would produce, but did correlate with the estimated number of "significant" fibres.

Stanton and Wrench (1972) also produced mesotheliomata with very fine fibreglass. Mesotheliomata were not produced by our sample of fibreglass but they were by synthetic aluminium silicate fibre, which was finer than our glass fibre (Fig. 7 and 8). Again, the apparent contradiction is explicable in terms of physical characterizations. The 2 samples of fibreglass can only be compared using light microscope size data; in our sample 55% of the fibres had diameters exceeding 2·5 $\mu$m compared with less than 10% of Stanton and Wrench's sample.

Although a direct association between physical factors and the development of mesotheliomata has not been demonstrated, these characteristics appear to be the relevant properties. If the finer fibres are the more carcinogenic when applied to the pleura then, since the finer fibres are also able to penetrate to the pleura more easily after inhalation, these 2 factors would combine together to give the finer fibres more relative importance than even the aerodynamic differences would suggest.

Clearly, the experiments described in this paper have involved a large amount of daily effort over a number of years and we are grateful to all our colleagues who have contributed to this. We are also grateful to Dr B. T. Commins of the MRC Air Pollution Unit who prepared the benzene-extracted samples and the barium sulphate sample.

### REFERENCES

Berry, G. & Wagner, J. C. (1969) The Application of a Mathematical Model Describing the Times of Occurrence of Mesotheliomas in Rats following Inoculation with Asbestos. *Br. J. Cancer*, **23**, 582.

Commins, B. T. & Gibbs, G. W. (1969) Contaminating Organic Material in Asbestos. *Br. J. Cancer*, **23**, 358.

Harington, J. S. (1962) Occurrence of Oils Containing 3 : 4-Benzpyrene and Related Substances in Asbestos. *Nature, Lond.*, **193**, 43.

Harington, J. S. & Roe, F. J. C. (1965) Studies of Carcinogenesis of Asbestos Fibres and their Natural Oils. *Ann. N.Y. Acad. Sci.*, **132**, 439.

Holmes, A., Morgan, A. & Sandalls, F. J. (1971) Determination of Iron, Chromium, Cobalt, Nickel and Scandium in Asbestos by Neutron Activation Analysis. *Am. industr. Hyg. Ass. J.*, **32**, 281.

Lee, P. N. & O'Neill, J. A. (1971) The Effect of Time and Dose Applied on Tumour Incidence Rate in Benzopyrene Skin Painting Experiments. *Br. J. Cancer*, **25**, 759.

Morgan, A. & Cralley, L. J. (1973) Chemical Characteristics of Asbestos and Associated Trace Elements. In *Proc. Conf. Biological Effects of Asbestos*. Lyon, 2–5 October, 1972. In print.

Pike, M. C. (1966) A Method of Analysis of a Certain Class of Experiments in Carcinogenesis. *Biometrics*, **22**, 142.

Pike, M. C. & Doll, R. (1965) Age at Onset of Lung Cancer: Significance in Relation to Effect of Smoking. *Lancet*, i, 665.

Roe, F. J. C., Walters, M. A. & Harington, J. S. (1966) Tumour Initiation by Natural and Contaminating Asbestos Oils. *Int. J. Cancer*, **1**, 491.

Stanton, M. F. & Wrench, C. (1972) Mechanisms of Mesothelioma Induction with Asbestos and Fibrous Glass. *J. natn. Cancer Inst.*, **48**, 797.

Timbrell, V. (1965) The Inhalation of Fibrous Dusts. *Ann. N.Y. Acad. Sci.*, **132**, 255.

Timbrell, V. (1973) Physical Factors as Aetiological Mechanisms. In *Proc. Conf. Biological Effects of Asbestos*. Lyon, 2–5 October, 1972. In print.

Timbrell, V., Gilson, J. C. & Webster, I. (1968) UICC Standard Reference Samples of Asbestos. *Int. J. Cancer*, **3**, 406.

Timbrell, V., Pooley, F. & Wagner, J. C. (1970) Characteristics of Respirable Asbestos Fibres. In *Pneumoconiosis. Proc. Internat. Conf.* Johannesburg, 1969. Ed. H. A. Shapiro. Cape Town: Oxford University Press. p. 120.

Timbrell, V. & Rendall, R. E. G. (1971) Preparation of the UICC Standard Reference Samples of Asbestos. *Powder Technol.*, **5**, 279.

Wagner, J. C. (1970) The Pathogenesis of Tumours following the Intrapleural Injection of Asbestos and Silica. In *Morphology of Experimental Respiratory Carcinogenesis. Proc. Conf.* Gatlinburg 13–16 May 1970. Ed. P. Nettesheim, M. G. Hanna Jr. and J. W. Deatherage Jr. U.S. Atomic Energy Commission Symposium Series No. 21. p. 347.

Wagner, J. C. (1972) The Significance of Asbestos in Tissue. In *Recent Results in Cancer Research*, Vol. 39. *Current Problems in the Epidemiology of Cancer and Lymphomas*. Ed. E. Grundmann and H. Tulinius. Berlin: Springer-Verlag. p. 37.

Wagner, J. C. & Berry, G. (1969) Mesotheliomas in Rats following Inoculation with Asbestos. *Br. J. Cancer*, **23**, 567.
WAGNER, J. C., BERRY, G. & TIMBRELL, V. (1970) Mesotheliomas in Rats Following the Intra-pleural Inoculation of Asbestos. In *Pneumoconiosis. Proc. Internat. Conf.* Johannesburg, 1969. Ed. H. A. Shapiro. Cape Town: Oxford University Press. p. 216.

### APPENDIX

The results have been analysed using the model given by Pike (1966) and shown to be valid for experiments of this type (Berry and Wagner 1969). The age-specific death rate of animals dying with a mesothelioma $t$ days after injection is taken as $ck(t\text{-}w)^{k-1}$ where $c$, $k$ and $w$ are constants. These 3 constants could be estimated separately for each treatment of each experiment but correlations between the estimates make them imprecise. In the original experiments, with a total of 417 mesotheliomata, it was shown that $k$ could be taken as 3 for all treatments but that $w$, the lapse period before any mesotheliomata occurred, varied with treatment; in particular the lapse period for amosite was found to be about 200 days longer than that for chrysotile and crocidolite but there was an isolated mesothelioma occurring with amosite after only 398 days. For the experiments being reported here, although the estimates of the lapse period vary widely over the different treatments, this wide range could be due to the imprecision of the estimates, and there is no strong evidence that it is invalid to use a common value. Also, there is no evidence of the lapse period being dependent on dose. Therefore, in the analysis the best estimates of $k$ and $w$ based on all our evidence were used; these are $w = 270$ and $k = 3{\cdot}25$. Even with all the data these estimates are not very precise; for example the pairs (300, 2·9) and (220, 3·8) would be acceptable, as also would the estimates (250, 3·0) which we used in our preliminary reports. However, these alternatives all lead to similar conclusions.

The constant $c$ was estimated for each of the treatment groups and this constant, which we will refer to as the " carcinogenicity factor ", serves as a single index summarizing the mesothelioma experience of each treatment group. It combines the information on the proportion of animals which developed a mesothelioma with the times after inoculation at which the mesotheliomata occurred. Also, the method of estimation eliminates mortality due to other causes, so that neither chance nor systematic variations in natural mortality between different groups will affect comparisons between these groups.

As an example of the elimination of natural mortality, the proportion of rats developing mesotheliomata after injection with SFA chrysotile in Experiments 2 and 4 (69% and 64%) were similar to the proportion (65%) in SPF rats in our original experiment (Wagner and Berry, 1969). However, the rats in Experiments 2 and 4 were injected at 13 weeks of age opposed to 6 weeks in our original experiment, and also the natural mortality was less in our original experiment, even after allowing for this age difference. Hence the carcinogenicity of the chrysotile was least in our original experiment and this is reflected in the values of the carcinogenicity factor which were $1{\cdot}7 \times 10^{-9}$ in the original experiment, $4{\cdot}7 \times 10^{-9}$ in Experiment 2 and $2{\cdot}9 \times 10^{-9}$ in Experiment 4 (Table V). The non-significant difference between the values for Experiments 2 and 4 was revealed after eliminating the chance lower natural mortality in the group used in the latter experiment.


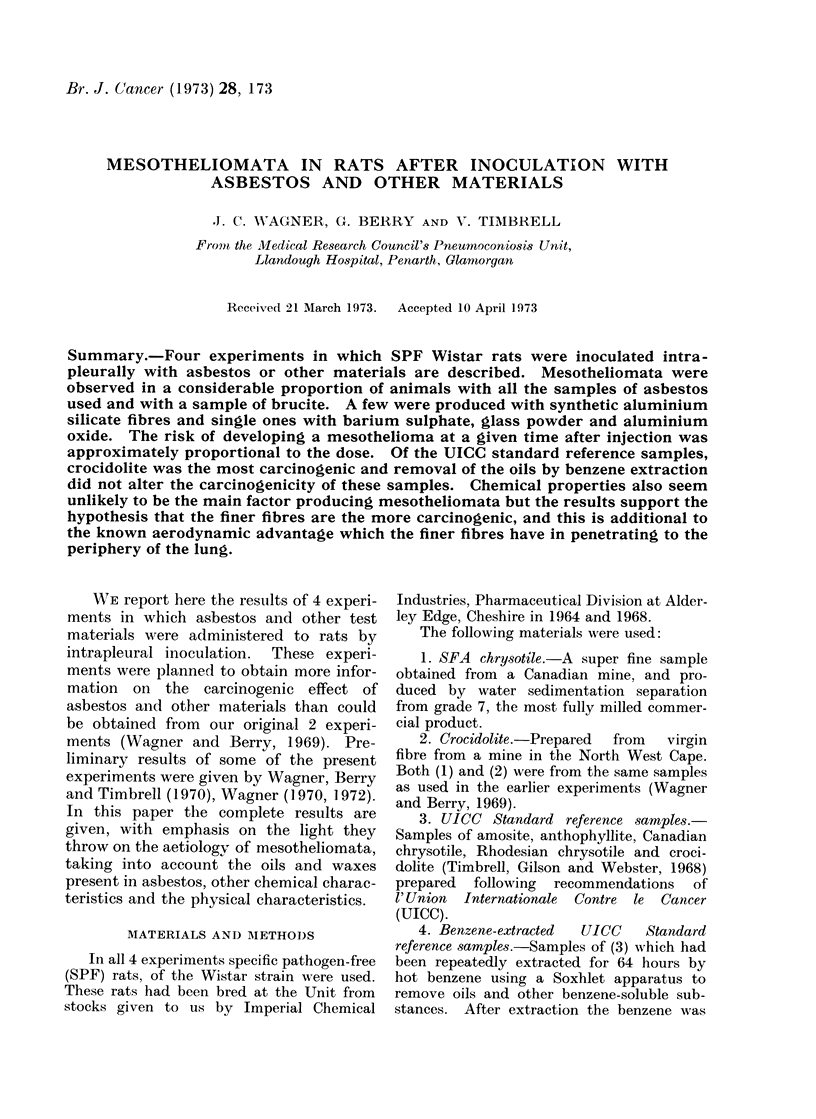

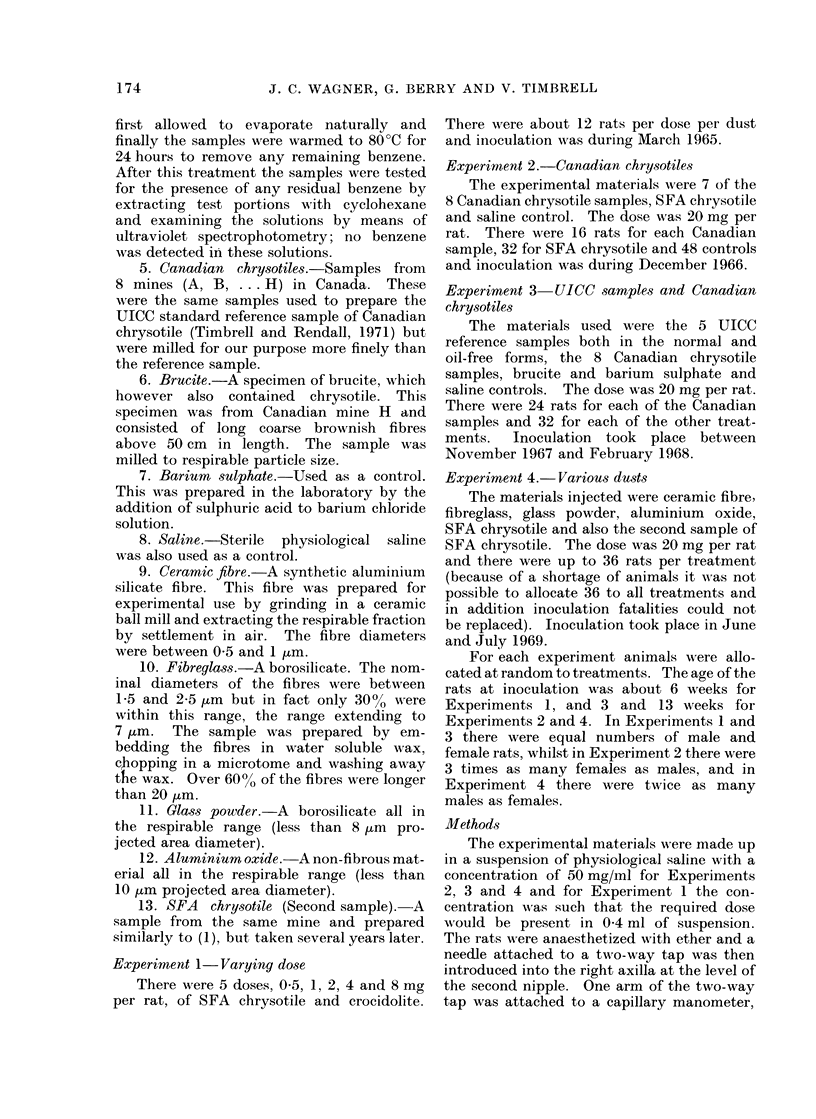

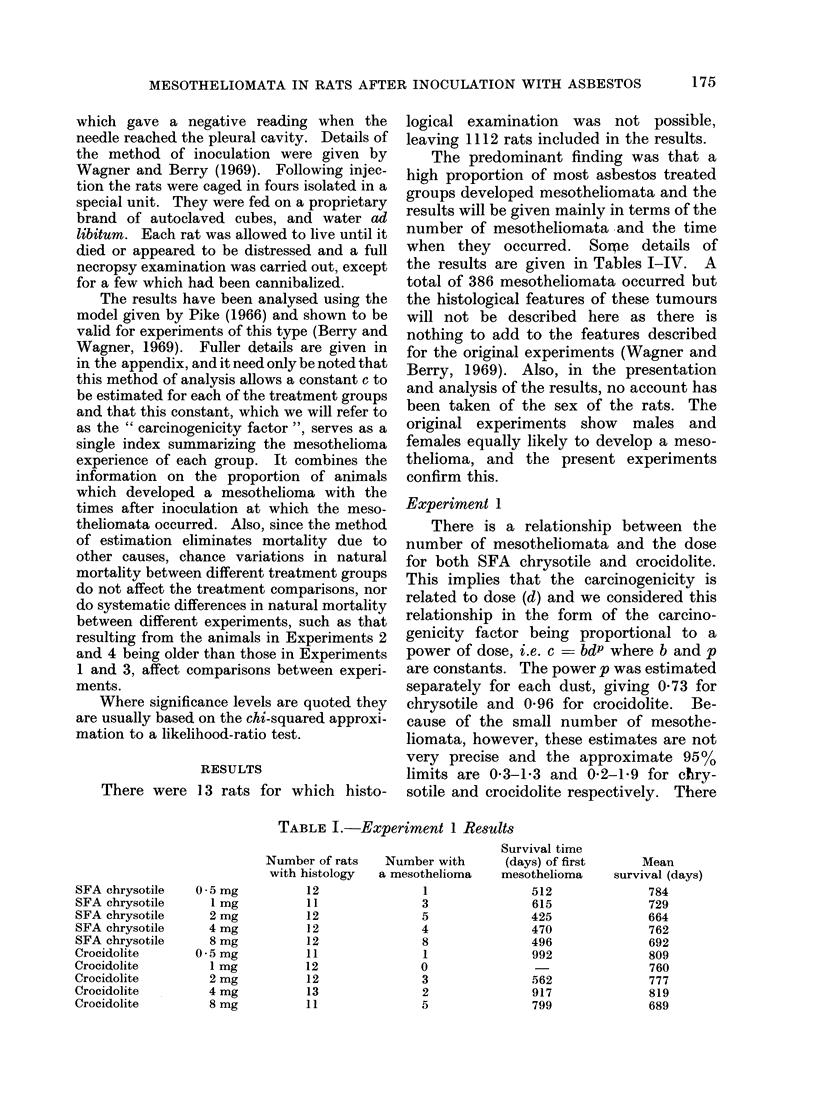

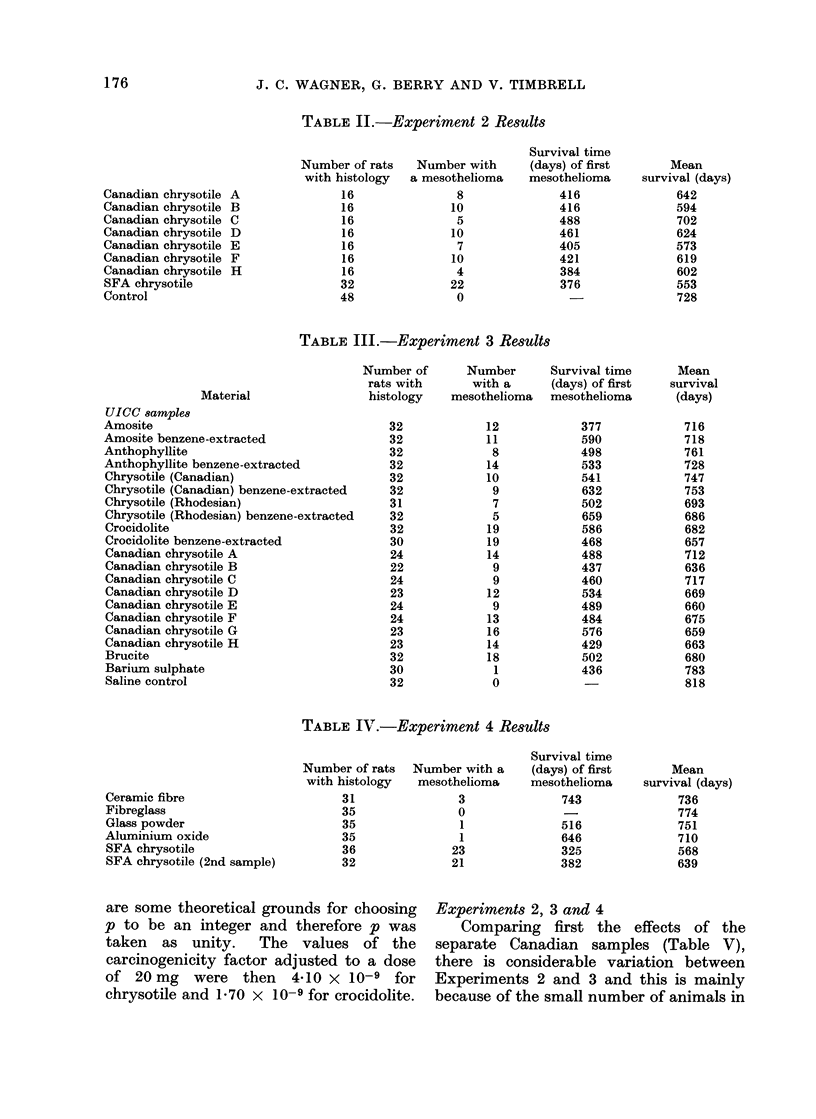

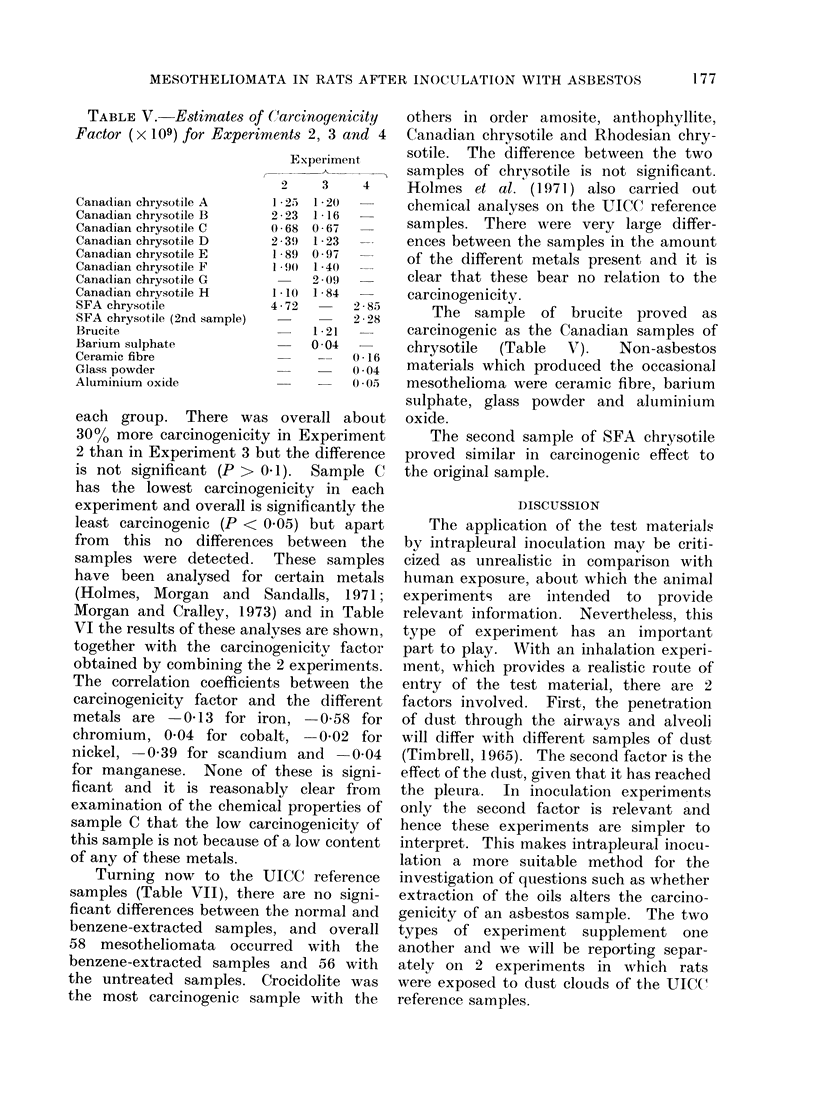

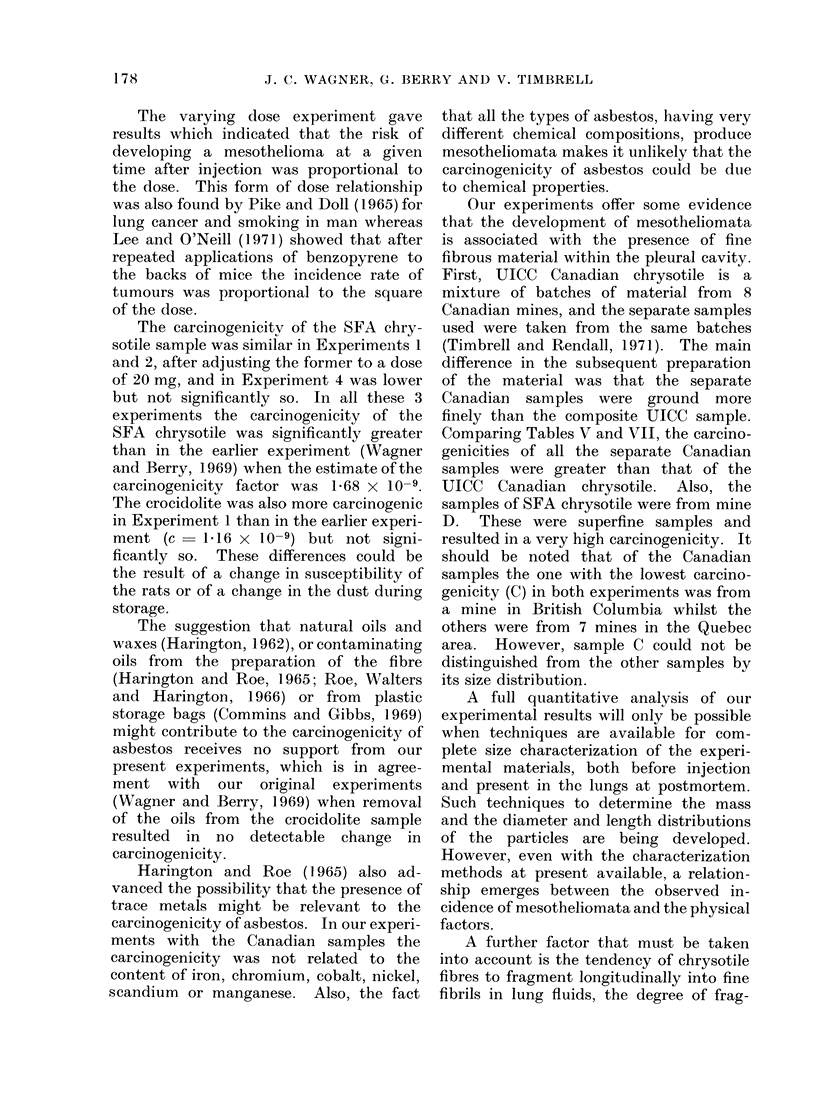

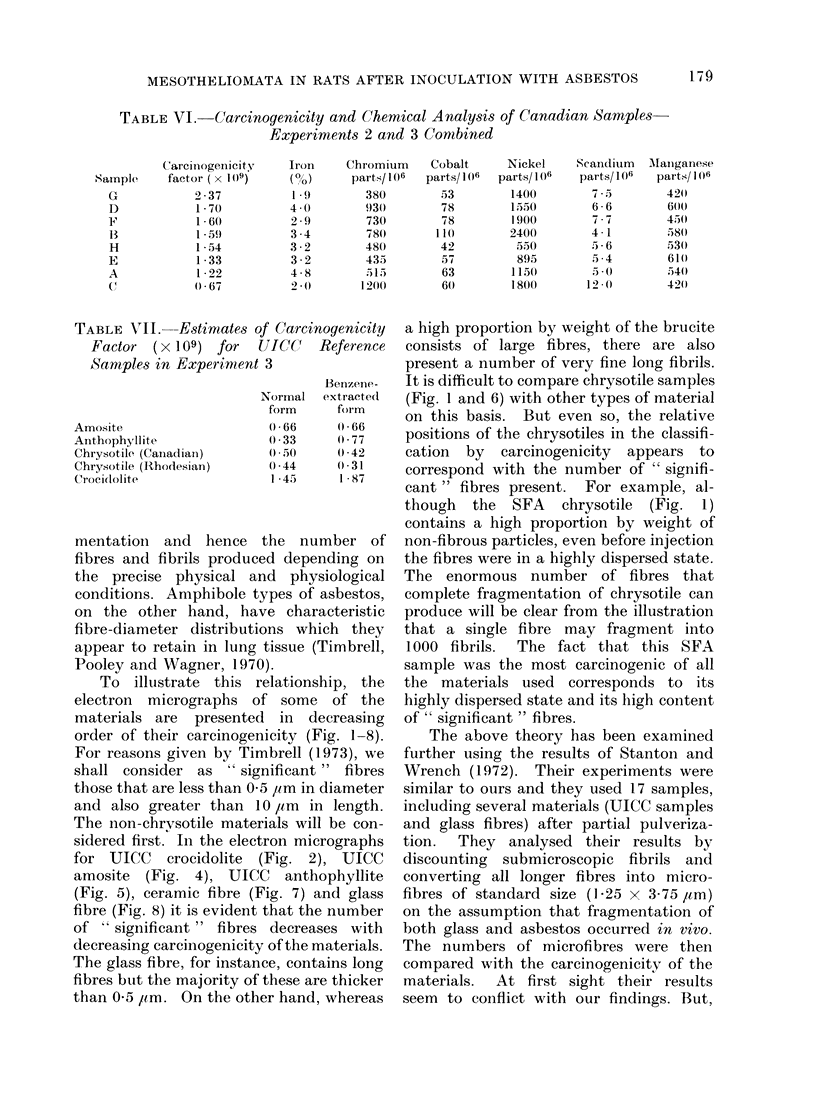

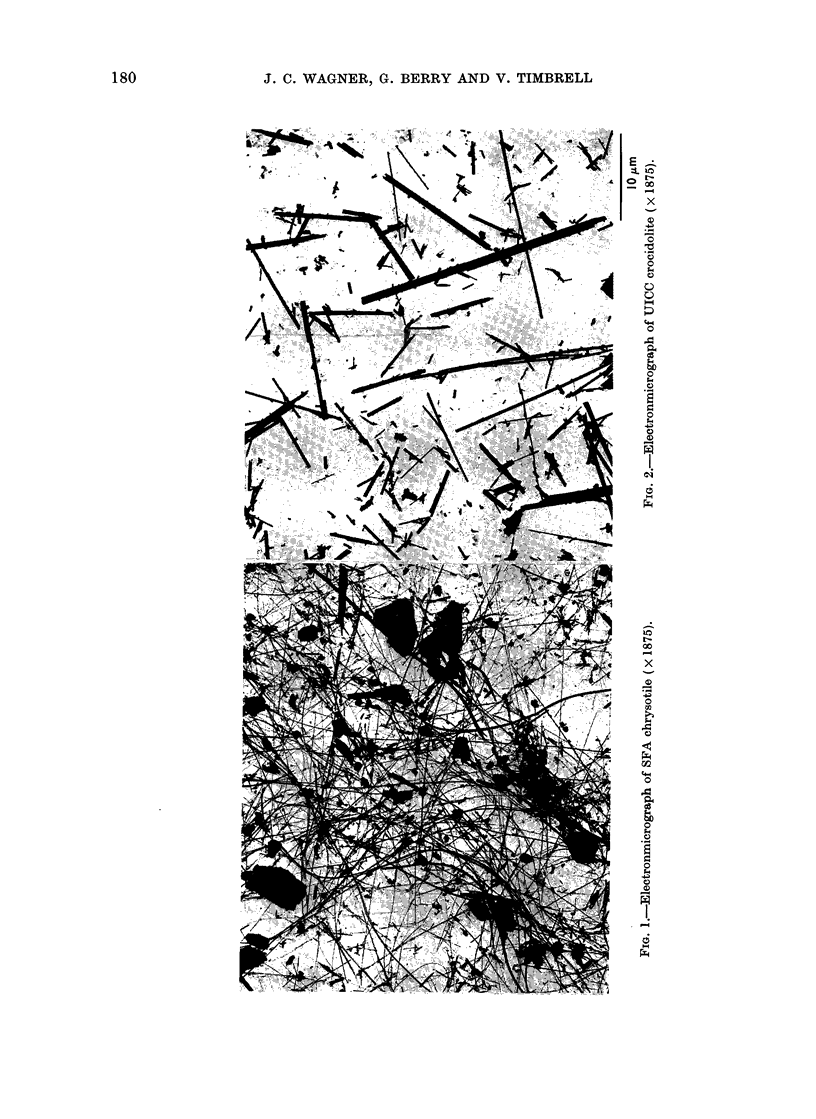

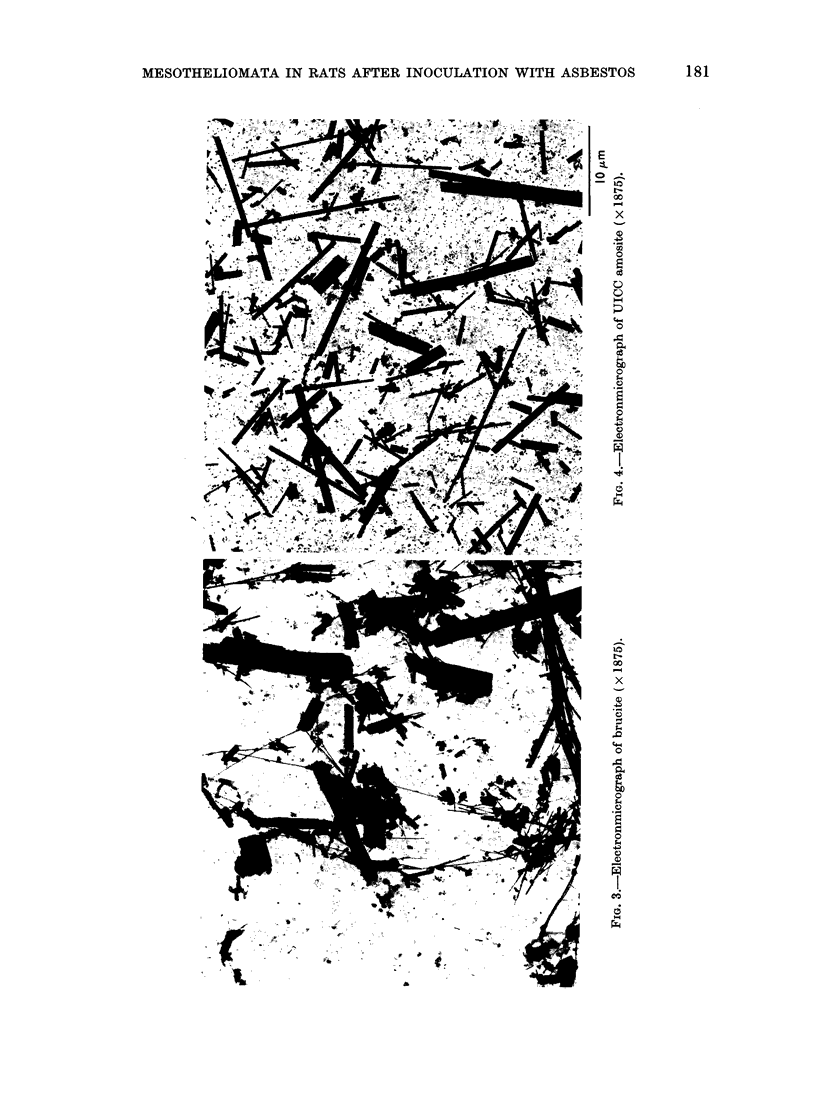

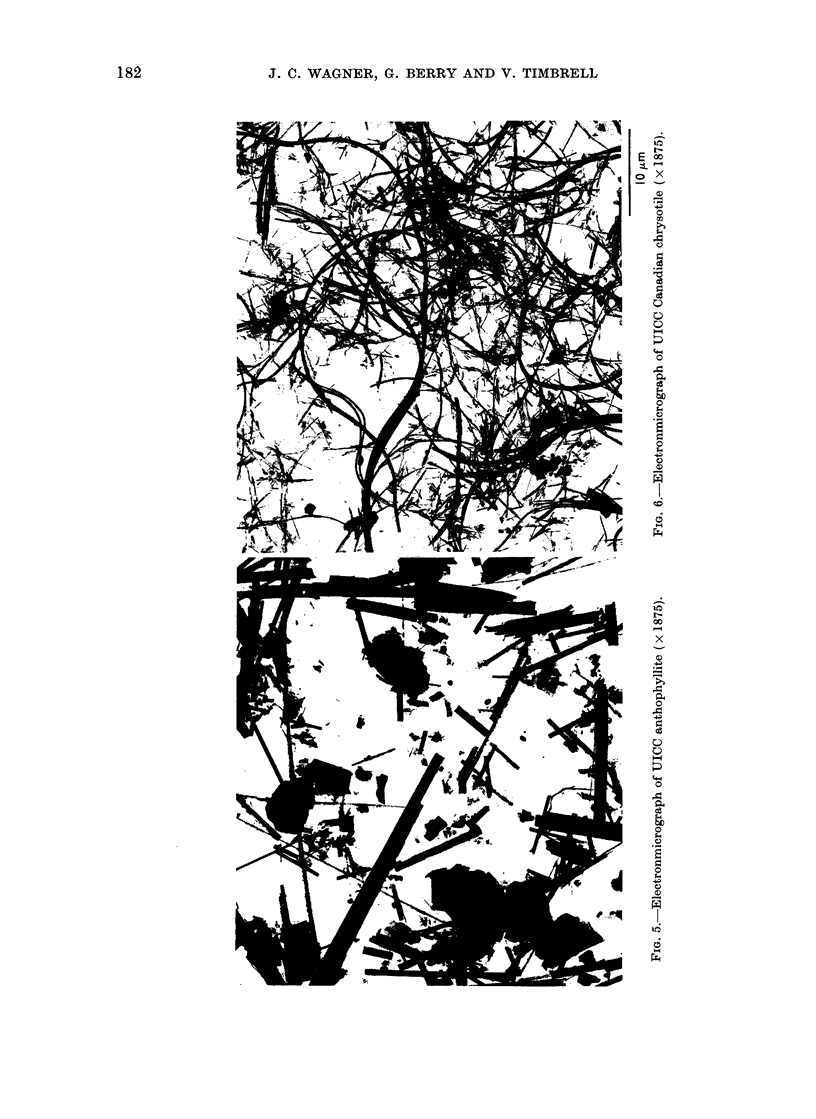

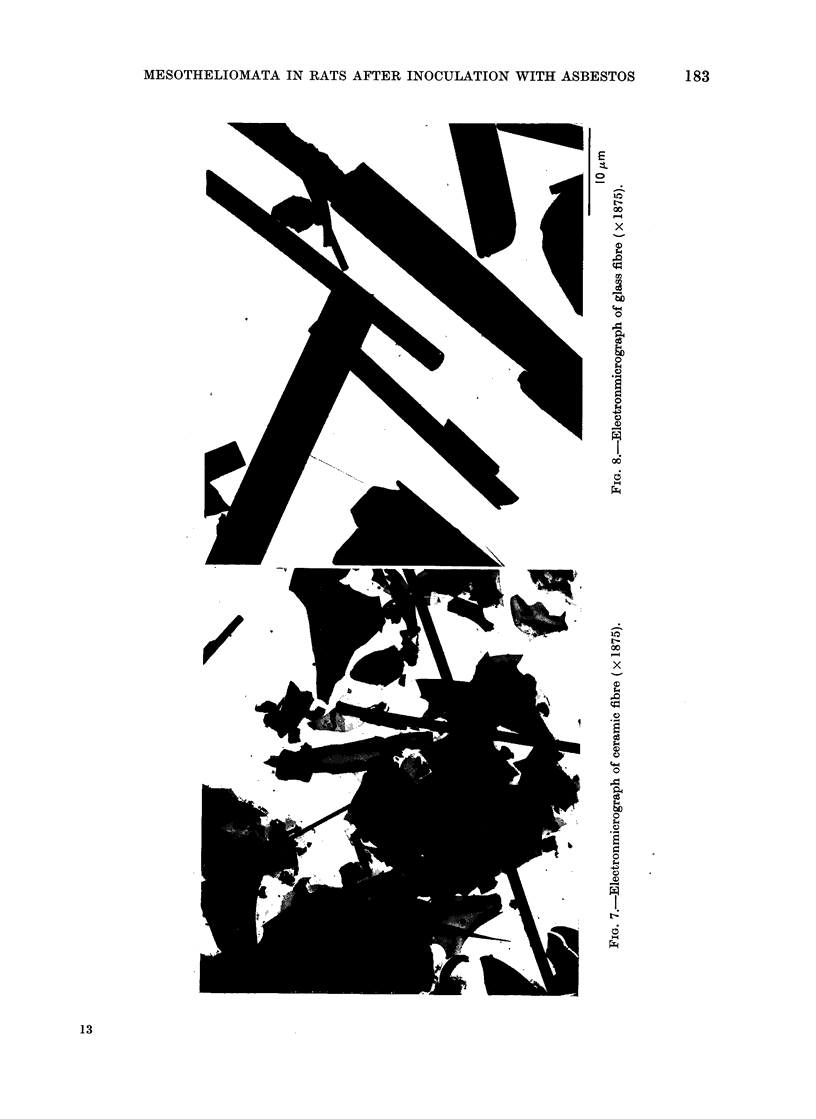

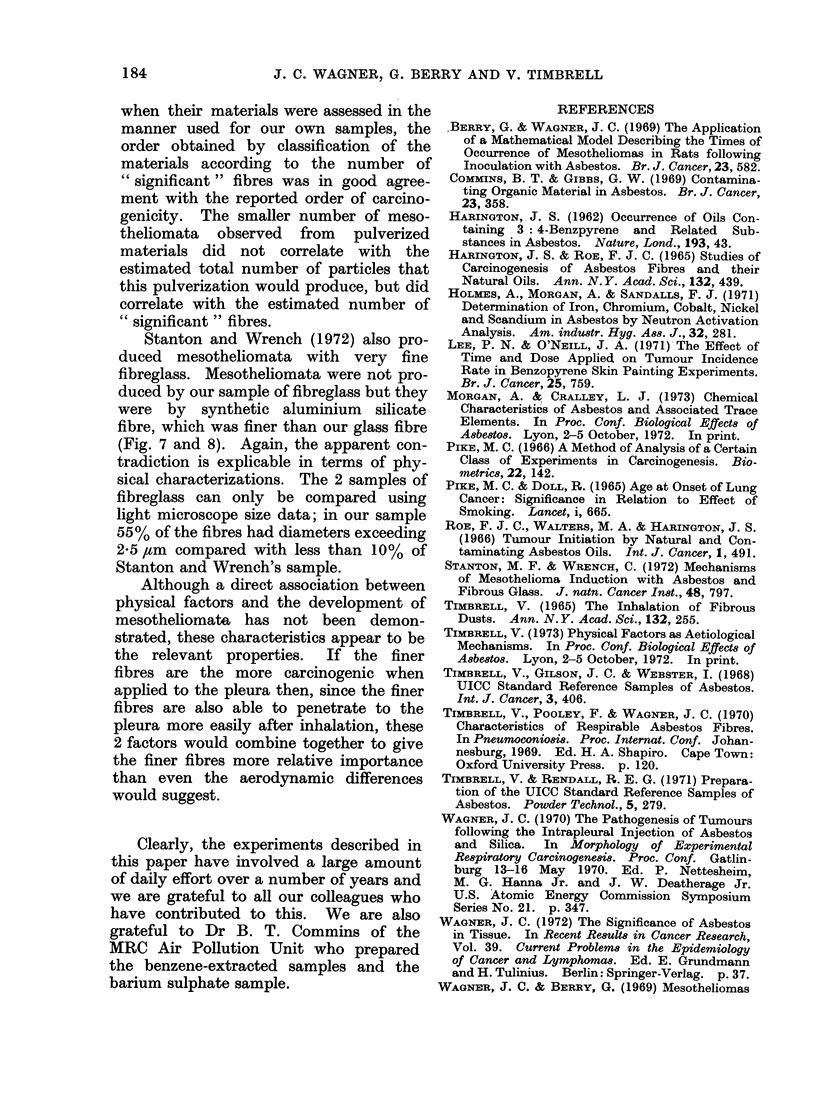

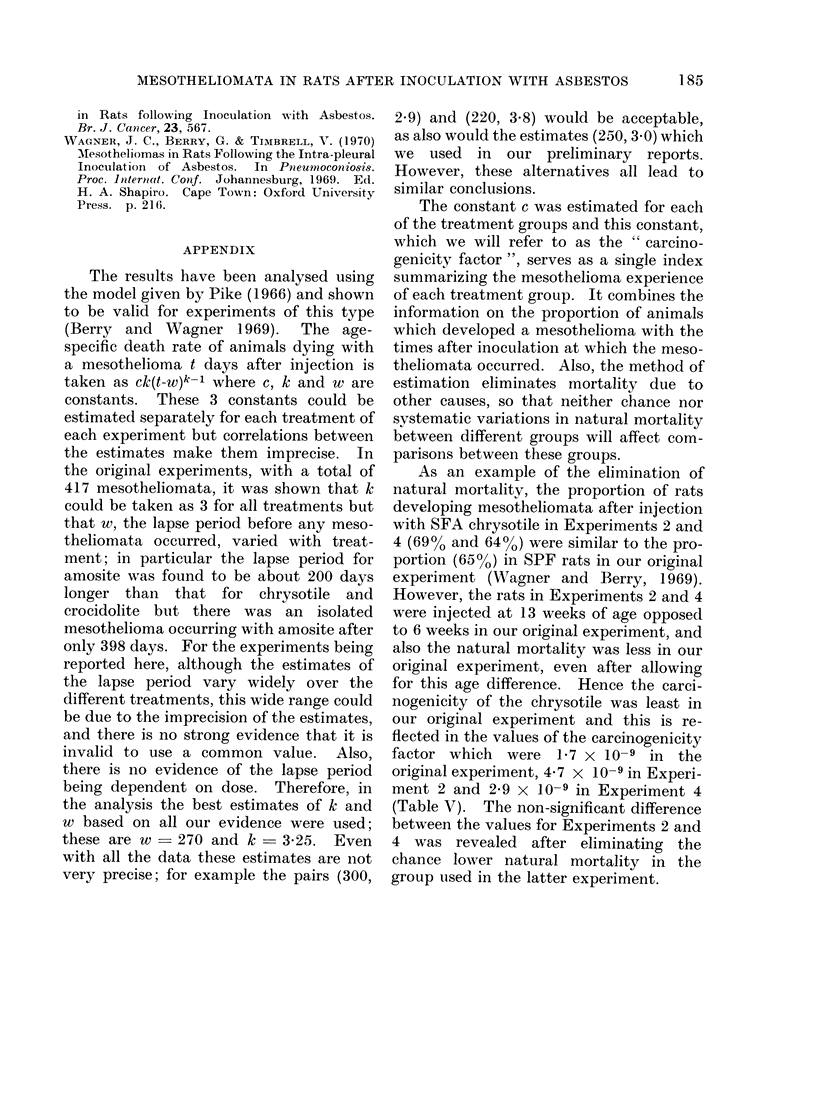


## References

[OCR_01196] Berry G., Wagner J. C. (1969). The application of a mathematical model describing the times of occurrence of mesotheliomas in rats following inoculation with asbestos.. Br J Cancer.

[OCR_01201] Commins B. T., Gibbs G. W. (1969). Contaminating organic material in asbestos.. Br J Cancer.

[OCR_01211] Harington J. S., Roe F. J. (1965). Studies of carcinogenesis of asbestos fibers and their natural oils.. Ann N Y Acad Sci.

[OCR_01216] Holmes A., Morgan A., Sandalls J. (1971). Determination of iron, chromium, cobalt, nickel, and scandium in asbestos by neutron activation analysis.. Am Ind Hyg Assoc J.

[OCR_01222] Lee P. N., O'Neill J. A. (1971). The effect both of time and dose applied on tumour incidence rate in benzopyrene skin painting experiments.. Br J Cancer.

[OCR_01239] PIKE M. C., DOLL R. (1965). AGE AT ONSET OF LUNG CANCER: SIGNIFICANCE IN RELATION TO EFFECT OF SMOKING.. Lancet.

[OCR_01234] Pike M. C. (1966). A method of analysis of a certain class of experiments in carcinogenesis.. Biometrics.

[OCR_01244] Roe F. J., Walters M. A., Harington J. S. (1966). Tumour initiation by natural and contaminating asbestos oils.. Int J Cancer.

[OCR_01248] Stanton M. F., Wrench C. (1972). Mechanisms of mesothelioma induction with asbestos and fibrous glass.. J Natl Cancer Inst.

[OCR_01262] Timbrell V., Gibson J. C., Webster I. (1968). UICC standard reference samples of asbestos.. Int J Cancer.

[OCR_01253] Timbrell V. (1965). Human exposure to asbestos: dust controls and standards. The inhalation of fibrous dusts.. Ann N Y Acad Sci.

